# Out-of-sample extension of diffusion maps in a computer aided diagnosis system. Application to breast cancer virtual slide images

**DOI:** 10.1186/1746-1596-8-S1-S9

**Published:** 2013-09-30

**Authors:** Belhomme Philippe, Oger Myriam, Michels Jean-Jacques, Plancoulaine Benoît

**Affiliations:** 1 BioTICLA-HIQ EA 4656, Université de Caen Basse-Normandie, Caen, France; 2 BioTICLA-HIQ EA 4656, CLCC François Baclesse, Caen, France

## Introduction

While the pathologist population tends to dramatically drop, the number of pathological cases to be examined increases sharply, mainly due to early screening campaigns; developing automated systems would thus be useful to help pathologists in their daily work. As Virtual Microscopy (VM) is more and more introduced in pathology departments [1] where it holds immense potential despite the large amounts of data to be managed, its combination with image processing techniques can allow to find objective criteria for differential diagnosis or to quantify prognostic markers. Thus, many works try to develop computer-aided diagnosis systems (CADS) based on image retrieval and classification [2,3]. The first step consists in building a knowledge database involving many features extracted from a set of well-known images; it is an 'off-line' procedure conducted once. These features are represented by vectors of non-linear data acting as a signature for the original images. In a second step, signatures are obtained from new unknown images to analyze and compared with the database; it is an 'on-line' procedure. Because of tumor heterogeneity, it is essential to build knowledge databases containing representative features of the multiple morphological types of lesions before considering to implement a CADS. But, as it is almost impossible for a pathologist to manually segment large virtual slide images (VSI), the usual practice consists in manually selecting some 'representative areas'. A bias is then introduced in the process as this choice is obviously subjective. It is then mandatory to find wiser solutions leading to an unbiased collection of these 'representative areas' (and later called 'patches'). In a previous work [4], we have proposed an original strategy: starting from a collection of breast cancer VSI, then taking advantage of stereological sampling methods and diffusion maps, a knowledge database is obtained from a reduced number of patches that are representative of given histological types. The sampling tools offered by stereology are well-suited in this context [5]. Systematic sampling starting from a random point with a fixed periodic interval is able to reduce the area to be analyzed, while preserving the collection of distinctive regions encountered in a tumor. However, even if the working area becomes smaller, the number of selected patches can be very large and may include many redundant elements. A data reduction has then to be conducted. Among the available methods, the diffusion maps technique [6,7] has been retained since it provides a very attractive framework for processing and visualizing huge non-linear bulk data. Diffusion maps belongs to unsupervised learning algorithms dealing with a spectral analysis of non-linear data, providing a clustering only for given training points with no straightforward extension for out-of-sample cases. The work presented here focuses on a way to get around this problem and explains how unknown VSI can be classified by considering the diffusion maps as a learning eigenfunction of a data-dependent kernel. It makes use of the Nyström formula to estimate diffusion coordinates of new data [8]. An application on histological types of breast cancer is presented with VSI of Invasive Ductal Carcinoma and Mastosis.

## Materials

VSI come from histological sections of breast tumors stained in the same laboratory according to the Hematoxylin-Eosin-Safron protocol and acquired with the same digital scanner (a ScanScope CS from Aperio Technologies). The aim being to develop a generalized CADS, it is mandatory to manage color calibration of each device used along the process, from histological staining up to image acquisition [9]. For this study, we have collected image patches from two histological types: Invasive Ductal Carcinoma (IDC) and Mastosis (Ma) with patches from the 'normal' morphology for further be able to remove non-informative patches. VSI have been acquired at X20 (0.5 µm per pixel) and stored in TIFF 6.0 file format (compression 30%). The tools are developed in Python language with the help of specialized modules (PIL: Python Imaging Library, SciPy and mathplotlib).

## Methods

### Stereology

In order to reduce the expertise workload and to obtain a reliable ground truth, a stereological test grid for point counting is over-imposed onto VSI in the ImageScope viewer [10]. The grid step has been set to 1000 x 1000 pixels (3500 points in average per image). The pathologist has then to determine which histological class is associated with the local areas centered on grid points; 30 possibilities are proposed for breast tumors. A simple mark has to be drawn on a grid point in the overlay layer whose name corresponds to his choice. Each area is then extracted at the plain resolution and stored as an uncompressed TIFF image. These areas (also called 'patches') are squares of size 400 x 400 pixels. This size has been chosen according to the representative structures encountered in breast tumors and allows to expertise only 16% of a VSI.

### Features extraction

For each patch, some statistical features are computed and embedded in a vector with its histological type and its coordinates in the stereological grid. At this stage of the study, all features are obtained from global measurements on patches computed on *RGB* color components (reduced to 64 values) and from the two first components (*H*, *E*) of the color deconvolution specific to Hematoxylin and Eosin staining [11]. For any given component *X*, the computed features are: *X*, *X* reverse sorting, cumulative_*X*, 20%-40%-60%-80% quantiles of cumulative_*X*, mean_*X*, median_*X*, mode_*X*, Skewness_*X*, Kurtosis_*X*, PearsonModeSkewness_*X*, that is a total of 13 data. Three of them are themselves histograms with 64 values but will provide a single measure after computing the distance between two signatures.With the 5 components (*R*, *G*, *B*, *H*, *E*) 65 measures will be taken into account for a patch but 1010 values will be stored in its signature. Considering the sparse numerical range of features, the symmetric Kullback-Leibler distance has been retained for its ability to easily manage such values, while remaining fast to implement. The distance between two vectors *p_1_*,*p_2_* of length *n* is then defined by:

### Data reduction

This study aims to develop a CADS whose one component is a visualization tool showing relations between breast cancer images, stored in a knowledge database, and new images presented to the system. Typically, these relations may be expressed as a connected graph in a 3D space where we hope to find 30 distinctive clusters corresponding to histological types or sub-types. It is therefore mandatory to reduce dimensionality from n (65 dimensions in our example) to 3. The signatures being non linear data, it is not appropriate to perform a principal component analysis (PCA). Belkin [5] and Coifman [6] have shown that methods based on Spectral Connectivity Analysis (SCA) such as diffusion maps, involving eigenvalues and eigenvectors from a normalized graph Laplacian, are well suited to non linear data. Let *X*={*x_1_*,*x_2_*,...,*x_n_*} be a set of *n* patches that we estimate as a fully connected graph *G*, that means a distance function is computed for each pair {*x_i_*,*x_j_*}. A *n*x*n* kernel *P* is obtained from a Gaussian function whose coefficients are given by:

In fact, *p*(*x_i_*,*x_j_*) may be viewed as the transition kernel of the Markov chain on *G*. In other words, *p*(*x_i_*,*x_j_*) defines the transition probability for going from *x_i_* to *x_j_* in one time step. The eigenvectors Π_k_ of *P*, ordered by decreasing positive eigenvalues, give the practical observation space axes. It must be noticed that Π_0_ is never used since linked to eigenvalue ⌊=1 (i.e. the data set mean or trivial solution). Projection is then done along (Π_1_,Π_2_,Π_3_) for a 3D visualization. Choosing *∑* in *w*(*x_i_*,*x_j_*) is an empirical task which should permit a moderate decrease of the exponential; some works use the median value of all distances *D_KL_*(*x_i_*,*x_j_*) where other use the mean distance obtained from the k nearest neighbors of a subset of *X *[6].

### Out-of-sample Nyström extension

SCA techniques share one major characteristic that is to compute the spectrum of a positive definite kernel. It is known that the eigenvalue decomposition of a matrix *P* ∊ ℝ ^n×n^ can be computed no faster than *O*(*n*^3^); this limits SCA techniques to moderately sized problems [12]. Fortunately, the Nyström extension, originally applied for finding numerical solutions of integral equations, can be used to compute eigenvectors and eigenvalues of a sub-matrix formed by *m* columns of *P* randomly subsampled and then extended to the remaining *n-m* columns [8]. Given an *n*x*n* matrix *P* and an integer *m*<*n*. Let call *P*^(^*^m^*^)^ the matrix formed by *m* columns of *P* that is the graph Laplacian of a set *Y*⊂*X* with |*Y*|=*m*. *Y* is then a training set. The orthonormal matrix of eigenvectors *U*^(^*^m^*^)^ and their associated eigenvalues in a diagonal matrix *Ë*^(^*^m^*^)^ are classically obtained from *P*^(^*^m^*^)^ by solving: *P*^(^*^m^*^)^*U*^(^*^m^*^)^=*Ë*^(^*^m^*^)^*U*^(^*^m^*^)^. This step has to be run once and then may be considered as an 'off-line' procedure. The Nyström formula allows to obtain the approximate eigenvectors of all the set *X* by:

where *λ_i_*^(^*^m^*^)^ and *u_i_*^(^*^m^*^)^ are the *i*^th^ diagonal entry and *i*^th^ column of *Ë*^(^*^m^*^)^ and *U*^(^*^m^*^)^ respectively. *P_N_*_,_*_M_* is a *n*x*m* sub-matrix of the complete graph obtained from distances *w*(*x_i_*,*x_j_*). Its computation is an 'on-line' procedure having to be conducted for each new test set (*X\Y*). For a 3D visualization, the second to fourth columns are used (the first one being the trivial solution).

## Results and discussion

**Figure 1:**
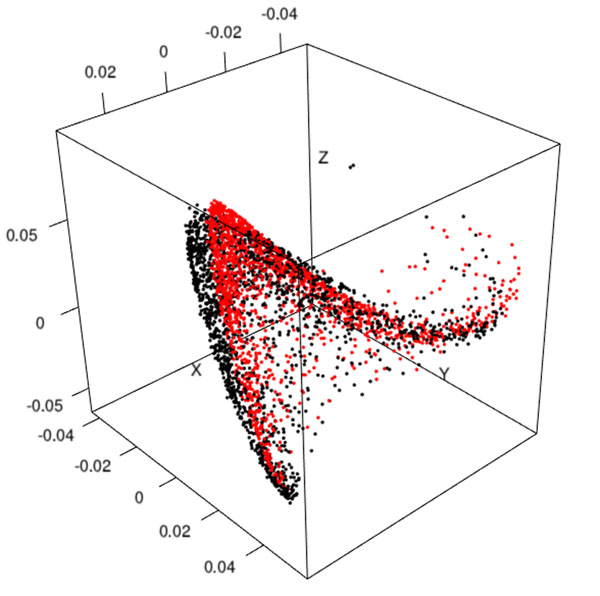
True eigenvectors coordinates (black) versus estimated coordinates (red) for 1000 test points.

**Figure 2:**
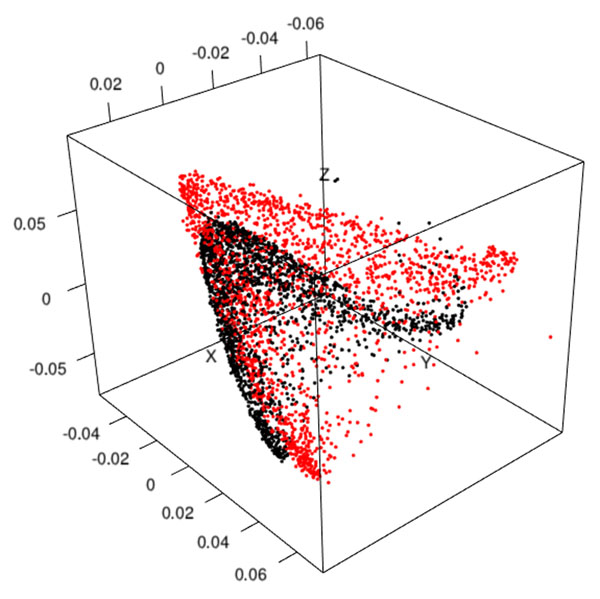
True eigenvectors coordinates (black) versus estimated coordinates (red) for 500 test points

To illustrate the out-of-sample extension to diffusion maps, 7 VSI of breast cancer cases have been used. Their mean size is 80 000 x 42 000 pixels^2^. A total number of 1857 patches, classified as Mastosis (919 Ma), Invasive Ductal Carcinoma (812 IDC) and Normal (126 Nor) have been extracted from their inner stereological test grid. At first, table 1 shows that features extraction is *O*(*n*) while the spectral analysis is close to *O*(*n^3^*); it has to be noticed that the latter involves both eigenvectors decomposition and code for managing the CADS. Figure 1 illustrates the projection of patches with their true eigenvectors (in black) and their estimated coordinates obtained from 1000 patches (in red). The visual comparison shows that computing a classical Euclidean distance between two points should be equivalent in both cases. Figure 2 shows the same approach from only 500 patches. Besides a shift between clouds of points, a rescaling is visible but the main shape is still preserved. To confirm this assertion we have analyzed for each patch the histological type of their nearest neighbor. This has been done both with the true eigenvectors and the estimated coordinates. In our application four cases are considered: a 'Ma' patch may be associated with another 'Ma' or 'IDC' whereas a 'IDC' patch may be associated with 'IDC' or 'Ma'. When a patch is close to the 'normal' type, we consider it as non-informative. Table 2 shows that the Nyström extension allows to obtain very similar results than the true eigenvectors (row 'reference').

**Table 1:** Computation time on a PC (dual core)

Patch number	Features extraction (in seconds)	Spectral analysis (in seconds)
250	46	17

500	98	69

1000	180	308

2000	407	1429

**Table 2:** Histological type in the nearest neighborhood

Number of test points	Ma		IDC	
	
	Ma	IDC	Ma	IDC
500	70.7%	18.0%	15.6%	78.8%

1000	72.2%	17.6%	15.3%	80.4%

reference	73.9%	17.1%	14.8%	82.1%

## Conclusion

This work is the second part of a CADS we aim to develop based on an original strategy starting from VS and leading to an unbiased knowledge database containing reference patches of breast tumors. The first part has been presented in [4]. We have shown that combining stereological sampling and data reduction based on diffusion maps offers an interesting general framework. The results illustrated here are a proof of concept of the second part that is to classify new unknown patches. About 400 high resolution VS are now available in our lab; the benign and malignant breast tumors are classified into 30 histological types and subtypes. We plan to project some reference patches extracted from these 30 classes in the same 3D space, in order to build clusters, and then to classify a new unknown VS previously split in patches. But the spectral decomposition is very CPU intensive and managing for example 30 000 patches at a time (1 000 per histological type) would rapidly become impossible to compute. The Nyström extension seems to provide a good approximation of eigenvectors which then allow to reduce this computational burden.
